# Malaria parasite detection and cell counting for human and mouse using thin blood smear microscopy

**DOI:** 10.1117/1.JMI.5.4.044506

**Published:** 2018-12-12

**Authors:** Mahdieh Poostchi, Ilker Ersoy, Katie McMenamin, Emile Gordon, Nila Palaniappan, Susan Pierce, Richard J. Maude, Abhisheka Bansal, Prakash Srinivasan, Louis Miller, Kannappan Palaniappan, George Thoma, Stefan Jaeger

**Affiliations:** a Lister Hill National Center for Biomedical Communications, National Library of Medicine, Bethesda, Maryland, United States; b University of Missouri-Columbia, Informatics Institute, Missouri, United States; c University of Colorado Boulder, Aerospace Engineering Sciences Department, Boulder, Colorado, United States; d National Institute of Allergy and Infectious Diseases, Division of Intramural Research, Rockville, Maryland, United States; e University of Missouri-Kansas City, School of Medicine, Kansas City, Missouri, United States; f University of Oxford, Centre for Tropical Medicine and Global Health, Nuffield Department of Medicine, Oxford, United Kingdom; g Mahidol University, Mahidol-Oxford Tropical Medicine Research Unit, Faculty of Tropical Medicine, Bangkok, Thailand; h Harvard University, Harvard TH Chan School of Public Health, Boston, Massachusetts, United States; i Jawaharlal Nehru University, School of Life Sciences, New Delhi, India; j National Institute of Allergy and Infectious Diseases, Laboratory of Malaria and Vector Research, Rockville, Maryland, United States; k Johns Hopkins Bloomberg School of Public Health, Molecular Microbiology and Immunology, Baltimore, Maryland, United States; l University of Missouri-Columbia, Department of Electrical Engineering and Computer Science, Columbia, Missouri, United States

**Keywords:** Automated malaria diagnosis, computational microscopy imaging, thin blood smears, red blood cell infection, cell segmentation and classification

## Abstract

Despite the remarkable progress that has been made to reduce global malaria mortality by 29% in the past 5 years, malaria is still a serious global health problem. Inadequate diagnostics is one of the major obstacles in fighting the disease. An automated system for malaria diagnosis can help to make malaria screening faster and more reliable. We present an automated system to detect and segment red blood cells (RBCs) and identify infected cells in Wright–Giemsa stained thin blood smears. Specifically, using image analysis and machine learning techniques, we process digital images of thin blood smears to determine the parasitemia in each smear. We use a cell extraction method to segment RBCs, in particular overlapping cells. We show that a combination of RGB color and texture features outperforms other features. We evaluate our method on microscopic blood smear images from human and mouse and show that it outperforms other techniques. For human cells, we measure an absolute error of 1.18% between the true and the automatic parasite counts. For mouse cells, our automatic counts correlate well with expert and flow cytometry counts. This makes our system the first one to work for both human and mouse.

## 1 Introduction

Malaria is caused by parasites transmitted via bites of female Anopheles mosquitoes. Parasite-infected red blood cells (RBCs) lead to symptoms, such as fever, malaise, seizures, and coma, in severe cases. Fast and reliable diagnosis and early treatment of malaria is one of the most effective ways of fighting the disease, together with better treatments and mosquito control.^1^ Over half of all malaria diagnoses worldwide are done by microscopy^1^^,^^2^ during which an expert slide reader visually inspects blood slides for parasites.^3^^–^^5^ This is a laborious and potentially error-prone process, considering that hundreds of millions of slides are inspected every year all over the globe.^6^ Accurate parasite identification is essential for diagnosing and treating malaria correctly. Parasite counts are used for monitoring treatment effect, testing for drug-resistance, and determining disease severity. However, microscopic diagnostics is not standardized and depends heavily on the experience and expertise of the microscopist. A system that can automatically identify and quantify malaria parasites on a blood slide would offer several advantages: it would provide a reliable and standardized interpretation of blood films and reduce diagnostic costs by reducing the workload through automation. Further image analysis on thin blood smears could also aid discrimination between different species and identification of Plasmodium parasite life stages: rings, trophozoites, schizonts, and gametocytes.^2^^,^^7^^,^^8^

Although both thick and thin blood smears are commonly used to quantify malaria parasitemia, many of the computer-assisted malaria screening tools currently available rely on thin blood smears.^2^^,^^7^^,^^9^ Thick smears are mainly used for rapid initial identification of malaria infection but it can be challenging to quantify parasites, where the parasitemia is high, and to determine species.^10^^–^^20^ On thin smears, parasite numbers per microscopy field are lower and individual parasites are more clearly distinguishable from the background allowing more precise quantification of parasites and distinction between different species and parasite stages.^21^^–^^32^

We present an end-to-end automated detection system for identifying and quantifying malaria parasites (P. falciparum) in thin blood smears of both human and mouse. The main difference between human and mouse malaria parasites is that in mice, all the stages of the parasite can be seen in the peripheral blood, whereas in humans, the mature stages, such as trophozoites and schizonts, are mostly sequestered. Another difference is that P. falciparum has elongated, banana-shaped gametocytes and takes around 10 to 12 days until complete maturation, whereas the gametocytes in mouse are round and maturate faster. This makes our software robust to different visual patterns of parasite stages. In resource-limited settings, where research labs have no access to flow cytometry or other cell counting means, our software can help expedite research experiments on mice models, taking the manual cell counting load from researchers. Moreover, flow cytometry is too expensive for field-use and requires a technical person to prepare, acquire, and analyze samples.

**Fig. 1:**
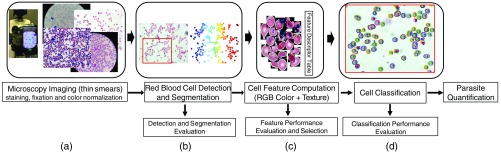
Our microscopy image analysis pipeline for counting malaria parasites. (a) Microscopy imaging of blood slides using a standard light microscope with a top-mounted camera or smartphone. (b) RBC detection and segmentation for thin smears. Evaluation will be performed in a standalone test-bed to compute the precision and accuracy of the cell detection and segmentation. (c) Extraction of segmented cells and computation of feature descriptors. The most discriminative features are selected through an offline evaluation framework. (d) Labeling detected cells into infected and uninfected using classification methods including SVM and ANN. Classification performance is evaluated separately to compute overall system precision, recall, accuracy, and F1 score.

Our automated malaria parasite detection system consists of four main steps, as illustrated in Fig. 1. In the first step, we prepare the blood slides by applying staining and fixation before collecting digitized images using a standard light microscope with a top-mounted camera [Fig. 1(a)].

We develop an efficient RBC detection and segmentation technique that uses a multiscale Laplacian of Gaussian (LoG) cell detection method as input to an active contours-based segmentation scheme named coupled edge profile active contours (C-EPAC) to accurately detect and segment individual RBCs and highly overlapping cells with varying annular and disk-like morphologies and textural variations [Fig. 1(b)]. Ersoy et al.^33^ presented C-EPAC to detect and track RBCs in videos of blood flow in microfluidic devices under controlled oxygen concentration. In this work, we evaluate the performance of C-EPAC on stained blood slides for malaria diagnosis that is new since RGB blood slide images have an entirely different characteristic than blood flow videos and accurate segmentation is essential to a successful cell classification. Furthermore, the iterative voting-based cell detection method that is used in C-EPAC is computationally expensive, which makes it not suitable for real-time processing. We use the multiscale LoG filter to detect cells, where local extrema of the LoG response indicate the approximate centroids of the individual cells. This provides us with a high cell detection accuracy and fast processing.

Then, we use a combination of color and texture features to characterize segmented RBCs. We develop an offline feature evaluation framework using manually annotated cells to select the most discriminative features, reduce feature dimensionality, and improve classification performance [Fig. 1(c)]. The feature evaluation results show that the combination of normalized red green blue (NRGB) color information and joint adaptive median binary pattern (JAMBP) texture features^34^ outperforms the other color models and texture features. The color model picks up the typical color information of stained parasites but is sensitive to lab staining variations. Therefore, we add the complementary JAMBP texture feature, which is invariant to staining variations, so that we can detect the distinctive cell texture information including the cytoplasm of parasites.

Finally, we use a linear support vector machine (SVM) to classify infected and uninfected cells because of its simplicity, efficiency, and easy translation to a smartphone [Fig. 1(d)]. We also evaluate and compare the SVM classifier results to an artificial neural network (ANN) classifier and demonstrate the comparable results.

The main contributions of this work are summarized as follows:

• The fusion of LoG filter with C-EPAC enables us to efficiently detect and segment individual RBCs, including highly overlapping cells with varying annular and disk-like morphologies and textural variations. We achieve a superior cell detection F1 score of 94.5% and 95% for human and mouse respectively, including a better performance in splitting touching or overlapping cells. We compute Jaccard indices of 92.5% for human cells and 81% for mouse cells.
• We use a combination of low-level complementary features to encode both color and texture information of RBCs. Features are selected through an offline evaluation framework to optimize the classification performance using manually annotated cells.
• We are the first to present a robust system for both human and mouse blood smears, including evaluation of the overall system performance in terms of precision, recall, accuracy, and F1 score. For human, we measure an average absolute error of 1.18% between the true and the automatic parasite counts. For mouse, we are the first to compare automatic cell counts with flow cytometry counts, measuring a high correlation.
• On average, our system can process about 100  cells/s on low-power computing platforms. This amounts to 20 s for 2000 cells, a number typically counted by a microscopist. A trained microscopist would need 10 to 15 min to examine a blood slide with 2000 cells and would therefore be much slower.

We organize the remainder of the paper as follows: Sec. 2 describes our image acquisition procedure and ground truth annotation tool. Section 3 presents our cell detection and segmentation process, followed by the object-level and the pixel-level evaluation results. Feature performance evaluation and selection are discussed in Sec. 4. In Sec. 5, we evaluate SVM and ANN classification performances before we summarize the main results and conclude the paper.

## 2 Materials and Procedures

We use blood slide images for both human and mouse provided by the National Institute of Allergy and Infectious Diseases (NIAID) to evaluate our system. All experiments are approved by the NIAID Animal Care and Use Committee (NIAID ACUC). The approved Animal Study Proposal (Identification Number LIG-1E) adheres to the regulations of the Animal Welfare Regulations and Public Health Service Policy on Human Care and Use of Laboratory Animals.

### 2.1 Malaria Blood Smears

#### 2.1.1 Human malaria infections

Whole blood from Interstate Blood Bank was processed to remove all the white blood cells by passing it through SEPACELL R-500 II leukocyte reduction filter from Fenwall. The processed blood was used to culture Plasmodium falciparum *in vitro* in the conditioned media comprising of RPMI 1640. The culture was maintained in a mixed gas environment with 5% $O_{2}$, 5% ${\mathrm{CO}}_{2}$ balanced by nitrogen.

#### 2.1.2 Mouse malaria infections

C57BL/6 female mice (7 to 10 weeks old) were obtained from The Jackson Laboratories. Mice were infected with PbA by injecting i.p. $1\times 10^{6}$ PbA-infected RBCs obtained from infected C57BL/6 mice.

#### 2.1.3 Flow cytometry

Peripheral blood parasitemia was determined by flow cytometry using a modification of a previously described method.^35^ Briefly, blood was obtained from mouse tail veins, fixed with 0.025% aqueous glutaradehyde solution, washed with 2 mL PBS, resuspended, and stained with the following: the DNA dye Hoechst 33342 (Sigma) (8  μM), the DNA and RNA dye dihydroethidium (diHEt) (10  μg/mL), the pan C57BL/6 lymphocyte marker allophycocyanin (APC)-conjugated Ab specific for CD45.2 (BioLegend), and the RBC marker APCCy7-conjugated Ab specific for Ter119 (BD Pharmingen). Cells were analyzed on a BD LSRII flow cytometer equipped with UV (325 nm), violet (407 nm), blue (488 nm), and red (633 nm) lasers. Data were analyzed using FlowJo software (Tree Star Technologies). iRBCs were $\mathrm{CD}45.2^{-}$, $\mathrm{Ter}119^{+}$, ${\mathrm{Hoechst}}^{+}$, and ${\mathrm{diHEt}}^{+}$. Parasitemia was calculated as the number of iRBCs divided by the total number of RBCs.

In the following sections, we will refer to the acquired slide images and annotations as the human-NIAID and mouse-NIAID datasets since the human and mouse blood slides have been provided by NIAID.

### 2.2 Image Acquisition and Annotation

**Fig. 2:**
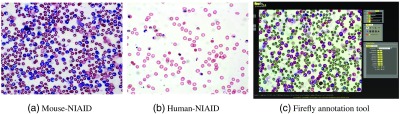
Examples of malaria images: (a) mouse-NIAID, (b) human-NIAID, and (c) labeling malaria microscopy slides using Firefly annotation tool: firefly.cs.missouri.edu

Blood slide images were acquired with the Zeiss Axio Imager, an upright research microscope platform, using a magnification of 63× and a standard Zeiss oil immersion lens. The dimension of the images is 1380×1040, for both human and mouse, in RGB color space. The average bounding box dimension of an uninfected RBC is 40×40. We used only a single imaging plane, and no focus stacking in particular. Figures 2(a) and 2(b) illustrate two sample images from our mouse and human malaria image datasets.

Cells were manually annotated by an expert as either infected or uninfected, using our Firefly online annotation tool [Fig. 2(c), firefly.cs.missouri.edu]. Firefly is a web-based ground-truth annotation tool for visualization, segmentation, and tracking. It allows point-based labeling or region-based manual segmentation. We used Firefly’s interactive fast point-based labeling to compute the actual infection ratio for each slide, which is computed as the ratio of the number of infected cells over the total number of cells in the slide. Furthermore, we used region-based manual segmentation of cells to evaluate cell detection and segmentation results.

We evaluate the performance of features and classifiers on 70 human-NIAID images (14  slides×5  images) and 66 images from mouse-NIAID dataset that were uploaded to Firefly and labeled pointwise by placing dots in different colors on infected and uninfected cells. Our mouse-NIAID dataset contains two sets of images named 2805 and 2808; each contains 33 images (11  slides×3  images). However, the cell boundaries annotations that are required for segmentation evaluation are available only for 10 images of human-NIAID and six images from mouse-NIAID, which are used for cell detection and segmentation evaluation. These are our so-called fully annotated images.

## 3 Automatic Detection and Segmentation of Red Blood Cells

RBCs detection and segmentation is the first challenging task in our malaria parasites detection pipeline, see Fig. 1(b).^2^^,^^36^^,^^37^ The main challenges are low image contrast, cell staining variations, uneven illumination, shape diversities, size differences, texture complexities, and particularly touching cells. Note that the accuracies of cell detection and segmentation directly affect the classifier performance; therefore, both have received much attention in the literature. Different techniques have been proposed including Otsu thresholding^12^^,^^38^^–^^40^ and watershed algorithms^41^^–^^43^ that are usually combined with morphology operations to improve segmentation results and address texture complexities; however, improper clump splitting and over-segmentation are the main drawbacks of the methods based on histogram thresholding and watershed transform.^7^ To address the splitting of overlapping cells and avoid over segmentation marker-controlled watershed algorithms,^25^^,^^44^^,^^45^ template matching,^32^^,^^46^ Ada-boost,^31^ distance transform,^47^ and active contour models^21^^,^^48^^–^^50^ have been presented, which perform poor to segment highly overlapping cells.

**Fig. 3:**
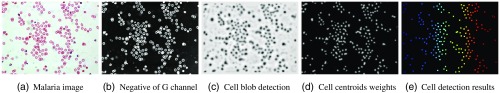
Illustration of cell detection results using Laplacian of Gaussian LoG filter. (a) Original malaria RGB color image, (b) negative of green channel being enhanced using histogram stretching, (c) local extrema of the LoG response that indicate the approximate centroids of the individual cells, (d) cell centroid weights using the distance transform of the cell foreground mask using Otsu thresholding, and (e) cell initial markers using weighted LoG blob responses.

In this paper, we fuse multiscale LoG filter withC-EPAC to efficiently detect and segment individual RBCs and highly overlapping cells with varying annular and disk-like morphologies and textural variations. C-EPAC^33^ is an extension of geodesic active contour models that enables robust cell segmentation particularly when RBC densities are high and touching cells are highly overlapping [Fig. 2(a)]. It begins with a voting-based cell detection scheme followed by a C-EPAC segmentation method. However, the iterative voting-based cell detection method is computationally expensive, which makes it not suitable for our real-time processing. We use the multiscale LoG filter to detect cells, where local extrema of the LoG response indicate the approximate centroids of the individual cells. Figure 3 illustrates the LoG-based cell detection method for a sample image from human-NIAID dataset. In the first step, we compute the negative of the green channel and enhance its contrast using histogram stretching (I^(x,y)). This makes the cells appear lighter than the background [Fig. 3(b)]. Then, we convolve the resulting image with the second derivative of Gaussian kernels over the x and y axis, and compute the Laplacian operators (L) at multiple scales $\sigma_{i}$:

Lxx(x,y,σi)=I^(x,y)*∂2∂x2G(x,y,σi),Lyy(x,y,σi)=I^(x,y)*∂2∂y2G(x,y,σi),L(x,y,σi)=Lxx(x,y,σi)+Lyy(x,y,σi), (1)

where $G(x,y,\sigma_{i})=\frac{1}{2\pi \sigma_{i}^{2}}e^{-\frac{x^{2}+y2}{2\sigma_{i}^{2}}}$. The local minima of L across scales indicate the approximate centroids of the individual cells [Fig. 3(c)]. In the last step, we weigh the LoG blob responses by the distance transform of the cell foreground mask [Fig. 3(d)] to generate cell initial markers [Fig. 3(e)]. This provides us with a high cell detection accuracy and meets the demands of real-time processing. After generating initial cell centroid markers, C-EPAC evolves a contour that starts from the centroids and expands to the precise boundaries of the cells. This method enables correct segmentation of both filled and annular cells by forcing the active contours to stop on specific edge profiles, namely on the outer edge of the RBCs. During contour evolution, multiple cells are segmented simultaneously by using an explicit coupling scheme that efficiently prevents merging of cells into clusters. The following section briefly reviews the C-EPAC level-set active contour-based segmentation algorithm.

### 3.1 C-EPAC Geodesic Active Contour Based Segmentation Algorithm

**Fig. 4:**
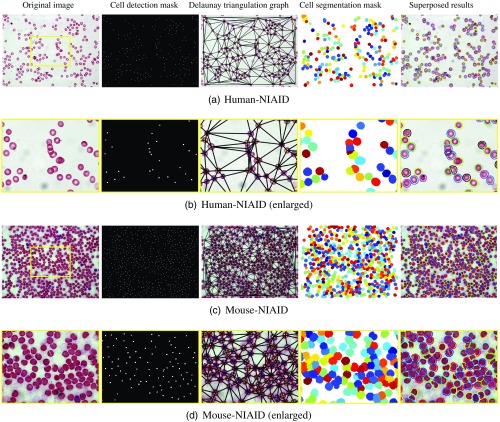
The illustration of cell detection and segmentation results for the combination of LoG with C-EPAC on a sample image from human-NIAID and mouse-NIAID. (a) Original image, (b) cell centroid detection mask, (c) Delaunay triangulation graph, (d) cell segmentation mask, and (e) cell segmentation contours superposed on the original image. The second and forth rows show the processing results enlarged for the region marked by the yellow box.

The regular geodesic active contour method usually suffers from early stops on irrelevant edges if not initialized properly. In order to obtain an accurate cell segmentation and prevent getting stuck at inner boundaries, C-EPAC^33^ is guided by a desired perpendicular edge profile I, which effectively lets the curve evolve through the inner boundary of the annular cell and stop at the correct outer boundary. The edge profile is computed as the intensity derivative in the direction of evolving surface normal, and the stopping function of C-EPAC, $g_{d}$, is defined as a decreasing function of the edge image gradient ∇I:

$$g_{d}(\nabla I)=1-H(-\frac{\nabla \phi }{\left|\nabla \phi \right|}\cdot \nabla I),\;H(x)=\{\begin{array}{cc} \begin{array}{l} 1\\ 0 \end{array} & \begin{array}{l} \text{if}\;x>0\\ \text{elsewhere} \end{array} \end{array},$$ (2)

where H is the Heaviside function and ϕ is the level set function. This sets $g_{d}$ to 1 at regions, where there is a bright-to-dark transition (inner contour of annular cell) perpendicular to the evolving level set, and to zero where there is a dark-to-bright transition (outer contour of annular cell). Thus, it lets the active contour evolve through the annular cells without getting stuck at the inner boundaries since both filled and annular cells have the same dark-to-bright profile on their outer boundaries in grayscale. The speed function of C-EPAC curve evolution is defined as follows:

$$\frac{\partial \phi }{\partial t}=(1-H(-\frac{\nabla \phi }{\left|\nabla \phi \right|}\cdot \nabla I))(F_{c}+K(\phi ))|\nabla \phi |+\nabla \phi \cdot \nabla [1-H(-\frac{\nabla \phi }{\left|\nabla \phi \right|}\cdot \nabla I)],$$ (3)

where t is time, $F_{c}$ is the constant balloon force that pushes the curve inward or outward based on its sign, and K is the curvature term. This approach can successfully find the precise boundaries of a single cell. However, when used for segmenting clustered RBCs, a single level set could produce a single, lumped segmentation by merging all contours that expand from several centroids. In order to avoid merging, C-EPAC considers an N-level set approach by utilizing the spatial neighborhood relationships between cells. The number of level sets (N) is equal to the number of colors that can be assigned to cells using a greedy graph coloring approach on the Delaunay graph of the spatial cell neighborhoods so that no two neighbor cells have the same color. We use six level set functions to cover all the cells in an image. Figure 4 illustrates the LoG cell detection results (second column) followed by C-EPAC segmentation results for two sample images from human-NIAID and mouse-NIAID datasets. The second and forth rows show the processing results enlarged for the region marked by the yellow box.

### 3.2 Red Blood Cell Detection and Segmentation Evaluation

In this section, we evaluate and compare the performance of our proposed cell detection and segmentation algorithm (LoG, C-EPAC) with the most popular methods including: (i) Otsu-thresholding combined with morphology operations (Otsu-M),^51^ (ii) marker control watershed algorithm (MCW),^52^ and (iii) Chan–Vese active contour cell segmentation approach (Chan–Vese).^53^

#### 3.2.1 Cell detection evaluation

**Fig. 5:**
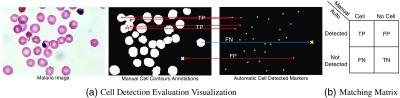
(a) Cell detection evaluation using a greedy algorithm that assigns each detected cell’s centroid (rightmost image) to its corresponding cell region in the ground-truth cell annotation mask (middle image). (b) Matching matrix.

The accuracy of cell segmentation relies on the performance of the cell detection algorithm in detecting individual and touching cells. Therefore, we first evaluate the performance of the LoG-based cell detection algorithm and compare it to cell detection results of Otsu-M and Chan–Vese active contour methods. Figure 5(a) illustrates our greedy cell detection evaluation approach for a sample human malaria microscopic image that assigns the automatic cell detection results (rightmost image) to its corresponding cell region in the ground-truth cell mask (middle image). We evaluate the performance of cell detection algorithms by computing the precision, recall, and F1 score using the matching matrix.^54^ The F1 score is the harmonic mean of precision and recall. The matching matrix is an error table, where each row represents the automatically detected cells and each column represents the manually detected cells given by ground-truth annotations [Fig. 5(b)]. Therefore, true positive (TP) is the cardinality of truly detected cells, false positive (FP) is the number of falsely detected cells, and false negative (FN) is the number of missed cells that are not automatically detected. In the context of cell detection evaluation, true negative (TN) is indeed the whole background region and is not considered in the calculation. Using the matching matrix allows us to compute the precision and recall performance of cell detection algorithms, where precision measures the rate of truly detected cells (TP) over the total number of automatically detected cells (TP+FP), and recall reports the rate of truly detected cells (TP) over the total number of cells using ground-truth annotations (TP+FN):

$$\text{Precision}=\frac{\left|\mathrm{TP}\right|}{\left|\mathrm{TP}+\mathrm{FP}\right|},\;\text{Recall}=\frac{\left|\mathrm{TP}\right|}{\left|\mathrm{TP}+\mathrm{FN}\right|}.$$ (4)

To combine the precision and recall performance of the detection algorithm and report the overall performance, F1 score is computed as follows:

$$F1=2\times \frac{\text{Precision}\times \text{Recall}}{\text{Precision}+\text{Recall}}.$$ (5)

Table 1 summarizes the cell detection performance evaluation results for LoG, Otsu-M, and Chan–Vese methods computed over 10 images from human-NIAID dataset that were fully annotated by an expert. Table 2 reports the cell detection evaluation results computed on six images from two slides from mouse-NIAID (for mouse 2805 and 2808). The precision, recall, and F1 score for LoG are clearly superior to Otsu-M and Chan–Vese active contour method, outperforming them by almost 4% on human-NIAID dataset and almost 8% on the mouse-NIAID dataset in terms of F1 score, when compared to Chan–Vese. MCW is not listed in Tables 1 and 2, because it relies on LoG cell detection method.

**Table 1:** Performance evaluation of LoG cell detection compared to Otsu-M and Chan–Vese methods for 10 images from human-NIAID dataset that were fully annotated by an expert. The reported precision, recall, and F1 score are the average results computed over 10 images and weighted by the number of cells per image.

Method	GT cells	Detected cells	TP	FP	FN	Precision	Recall	F1
Otsu-M	1460	1253	1242	11	218	0.991	0.851	0.915
Chan–Vese	1460	1237	1226	11	234	0.991	0.840	0.908
LoG	1460	1328	1318	10	142	0.993	0.903	0.945

**Table 2:** Performance evaluation of LoG cell detection compared to Otsu-M and Chan–Vese methods for six images from mouse-NIAID dataset that were fully annotated by an expert. The reported precision, recall, and F1 score are the average results computed over images and weighted by the number of cells per image.

Method	GT cells	Detected cells	TP	FP	FN	Precision	Recall	F1
Otsu-M	2446	2351	2157	194	289	0.920	0.882	0.900
Chan–Vese	2446	2145	2002	143	444	0.933	0.818	0.871
LoG	2446	2416	2304	112	142	0.954	0.944	0.949

#### 3.2.2 Cell segmentation evaluation

We compute the region-based Jaccard index^55^ for the four discussed segmentation algorithms Otsu-M, MCW, Chan–Vese, and C-EPAC algorithm to evaluate how accurately the RBC boundaries are detected.

Jaccard index is one of the most popular segmentation evaluation metrics that measures the similarity between a computed segmentation mask A and a ground-truth annotation mask B. The Jaccard similarity coefficient of two masks known as “intersection over union” is defined as follows:

$$\text{Jaccard}(A,B)=\frac{\left|A\cap B\right|}{\left|A\cup B\right|}=\frac{\mathrm{TP}}{\mathrm{TP}+\mathrm{FP}+\mathrm{FN}},$$ (6)

where TP is the number of pixels in A that are truly detected as cells, FP is the number of pixels in A that are falsely detected as cells, and FN is the number of pixels in B that are not detected as cells in A (missed).

**Fig. 6:**
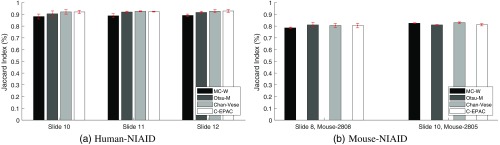
Cell segmentation performance evaluation of C-EPAC method compared to Otsu-M, MCW, and Chan–Vese on three slides from (a) human-NIAID and two slides from (b) mouse-NIAID dataset using Jaccard index. Each column represents the average of Jaccard index across three fields for each slide and method. The red vertical bar on top of each column shows the standard deviation.

Figure 6(a) shows the Jaccard index plotted for three slides from human-NIAID dataset, and Fig. 6(b) shows the two slides from mouse-NIAID dataset. Each column represents the average of Jaccard index across three fields for each slide and method. The red vertical bar on top of each column shows the standard deviation.

Figure 6(a) shows that our cell segmentation method provides a slightly better or similar performance for most of the fully annotated human-NIAID images achieving a weighted average of Jaccard Index of 92.5%. The computed weighted average of Jaccard index for Otsu-M is 91.4%, MCW is 88.4%, and 92.5% for Chan–Vese. For mouse cells, the computed weighted average of Jaccard index for Otsu-M is 80.4%, MCW is 81.0%, Chan–Vese is 81.7%, and 81.0% for C-EPAC [Fig. 6(b)].

**Fig. 7:**
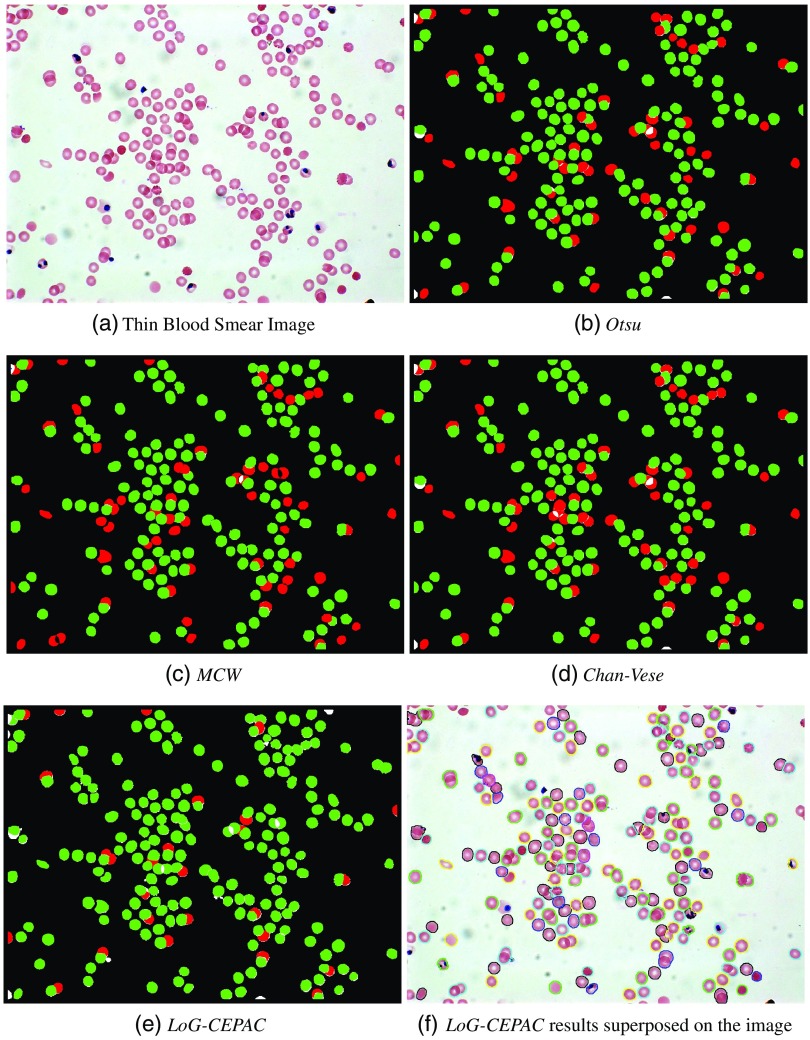
Illustration of cell detection and segmentation evaluation results using (a) thin blood smear image, (b) Otsu-M, (c) MCW, (d) Chan–Vese, (e) LoG-CEPAC, and (f) LoG-CEPAC, where green represents the truly detected cells (TP) and red represents missed cells (FN).

Figure 7 elaborates the cell detection and segmentation results for a sample image from human-NIAID dataset using the four discussed methods. From these figures, we conclude that our proposed LoG-CEPAC method provides superior results in efficiently detecting and segmenting individual RBCs and highly overlapping cells with varying annular and disk-like morphologies. Figure 7(f) shows our LoG-CEPAC results superposed on the original image. The combined performance improvements in cell detection and segmentation will lead to an overall performance improvement for practical applications.

## 4 Cell Feature Evaluation and Selection

**Fig. 8:**
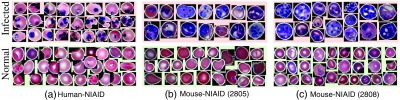
Examples of extracted infected (first row) and normal cells (second row) for (a) human-NIAID, (b) mouse-NIAID dataset 2805, and (c) mouse-NIAID 2808.

Once the cells have been detected and segmented from the whole image, in the next step, we extract all segmented cells and characterize them by their color and texture information to distinguish infected cells from normal cells within a learning framework. Figure 8 presents examples of extracted infected and normal cells (first and second rows) for human-NIAID (first column) and mouse-NIAID (second and third column) datasets.

We have studied different features for describing normal and abnormal cells, and evaluated their performance using SVM and ANN classifiers to select the most discriminative feature set. We evaluated the performance on both SVM and ANN to show that the best feature set outperforms other features independent from the classifier used. In feature evaluation experiments, we used ground truth annotations to extract cells and decouple the performance of features and classifier from our automatic segmentation results. The color feature set includes YCbCr, normalized green channel from RGB color model (NG), a combination of three discriminative normalized channels from different color models: $G_{\mathrm{RGB}}$, $S_{\mathrm{HSV}}$, $L_{\mathrm{LAB}}$ (NGSL), and NRGB. We also consider texture features to capture information about the appearance changes of the parasite during different stages of its life cycle in the human body. We evaluate the performance of linear binary pattern^56^ and JAMBP^34^ features. Table 3 lists the studied features and histogram descriptor’s dimension. For example, the NRGB is a composite of the three normalized color channels $R_{N}$, $G_{N}$, and $B_{N}$, each represented by a 16-bins histogram:

$$R_{N}=\frac{R}{R+G+B},\;G_{N}=\frac{G}{R+G+B},\;B_{N}=\frac{B}{R+G+B}.$$ (7)

**Table 3:** Features and dimensions.

Feature	Descriptor dimension	Remarks
YCbCr	10×3	Y is the luminance, Cb is the blue-difference and Cr is the red-difference chroma components.
LBP	18	Local binary pattern
NG	16	Normalized green channel
NGSL	16×3	Normalized green channel from RGB, saturation from HSV and L channel from LAB
NRGB	16×3	NRGB channel
NRGB + JAMBP	16×3+324	Combination of NRGB color and JAMBP texture features

To select the most discriminative feature set, we measure precision, recall, accuracy, and F1 score of SVM and ANN classifiers using the described features in Table 3. The accuracy is computed as follows:

$$\text{Accuracy}=\frac{\left|\mathrm{TP}+\mathrm{TN}\right|}{\left|\mathrm{TP}+\mathrm{FP}+\mathrm{TN}+\mathrm{FN}\right|}.$$ (8)

TP is the number of cells that are truly classified as infected and TN is the number of cells that are truly identified as normal cells. FP and FN report the number of cells that are being misclassified.

Tables 4 and 5 present the average performance evaluation results of SVM and ANN classifiers on 1615 manually segmented cells from the human-NIAID dataset using 10-fold cross-validation. The combination of color and texture features improves the F1 score of the SVM classifier from 88% to 93%, and the F1 score of the ANN classifier from 83% to 91%. Tables 6 and 7 report the same performance evaluation on 1551 manually segmented cells from the mouse-NIAID dataset using 10-fold cross-validation. An average high F1 score of 95% is achieved for the SVM classifier and 96% for the ANN classifier using the combination of NRGB and JAMBP. The tables show that a combination of NRGB and JAMBP performs well on both human and mouse datasets. Therefore, for every extracted cell, we compute a feature vector of size 372 including a 48-bins histogram of NRGB and a JAMBP texture feature vector of size 324.

**Table 4:** Feature performance evaluation using SVM classifier and ground-truth segmentation on human-NIAID.

${\mathrm{SVM}}_{\left(\text{human}\text{-}\mathrm{NIAID}\right)}$								
	TP	TN	FP	FN	Precision	Recall	Accuracy	F1
YCbCr	83	1489	37	6	0.69	0.93	0.97	0.79
LBP	73	1317	209	16	0.26	0.82	0.86	0.39
NG	86	1427	99	3	0.46	0.97	0.94	0.63
NGSL	71	1495	31	18	0.70	0.80	0.97	0.74
NRGB	81	1511	15	8	0.84	0.91	0.99	0.88
NRGB + JAMBP	83	1519	7	6	0.92	0.93	0.99	0.93

**Table 5:** Feature performance evaluation using ANN classifier and ground-truth segmentation on human-NIAID.

${\mathrm{ANN}}_{\left(\text{human}\text{-}\mathrm{NIAID}\right)}$								
	TP	TN	FP	FN	Precision	Recall	Accuracy	F1
YCbCr	67	1509	22	17	0.75	0.80	0.98	0.77
LBP	31	1514	58	12	0.35	0.72	0.96	0.47
NG	61	1500	28	26	0.69	0.70	0.97	0.69
NGSL	72	1507	17	19	0.81	0.79	0.98	0.80
NRGB	76	1508	13	18	0.85	0.81	0.98	0.83
NRGB + JAMBP	81	1517	8	9	0.91	0.90	0.99	0.91

**Table 6:** Feature performance evaluation using SVM classifier and ground-truth segmentation on mouse-NIAID.

${\mathrm{SVM}}_{\left(\text{mouse}\text{-}\mathrm{NIAID}\right)}$								
	TP	TN	FP	FN	Precision	Recall	Accuracy	F1
YCbCr	779	652	42	78	0.95	0.91	0.92	0.93
LBP	813	666	28	44	0.97	0.95	0.95	0.96
NG	757	620	74	100	0.91	0.88	0.89	0.90
NGSL	811	668	26	46	0.97	0.95	0.95	0.96
NRGB	817	662	32	40	0.96	0.95	0.95	0.96
NRGB + JAMBP	821	640	54	36	0.94	0.96	0.94	0.95

**Table 7:** Feature performance evaluation using ANN classifier and ground-truth segmentation on mouse-NIAID.

${\mathrm{ANN}}_{\left(\text{mouse}\text{-}\mathrm{NIAID}\right)}$								
	TP	TN	FP	FN	Precision	Recall	Accuracy	F1
YCbCr	794	620	63	78	0.93	0.91	0.91	0.92
LBP	774	607	83	91	0.90	0.89	0.89	0.90
NG	809	634	48	64	0.94	0.93	0.93	0.94
NGSL	796	630	61	65	0.93	0.92	0.92	0.93
NRGB	812	651	45	47	0.95	0.95	0.94	0.95
NRGB + JAMBP	828	653	29	45	0.97	0.95	0.95	0.96

## 5 Cell Classification and Labeling

In the last step of our processing pipeline, we use a SVM classifier with a linear kernel, a two-layer ANN feedforward network with a sigmoid transfer function in the hidden layer, and a softmax transfer function in the output layer to classify cells into two classes: infected and uninfected. We evaluate the system pipeline performance on a set of 14 thin blood slides, each containing 5 images, from human-NIAID dataset (for a total of 70 images and about 10,000 RBCs) using a 10-fold cross-validation scheme to train and test the classifiers. In each fold, 63 images are used for training and 7 images are used for testing.

Table 8 summarizes the average precision, recall, accuracy, and F1 score performance of the SVM and ANN classifiers. The SVM classifier achieves 98% accuracy in correctly identifying infected cells with a sensitivity (recall) of 91% and F1 score of 87%, which are comparable to the ANN classifier with 99% accuracy and F1 score of 90%.

**Table 8:** SVM and ANN classifiers performance evaluation using NRGB color and JAMBP texture features on 70 images from human-NIAID dataset.

Method	Cells	TP	TN	FP	FN	Precision	Recall	Accuracy	F1
ANN	8630	469	8054	62	45	0.88	0.91	0.99	0.90
SVM	8630	469	8018	98	45	0.83	0.91	0.98	0.87

To quantify the malaria infection, we compute the infection ratio as follows:

$$\text{Infection ratio}=\frac{\text{Number of infected cells}}{\text{Total number of cells}}.$$ (9)

**Fig. 9:**
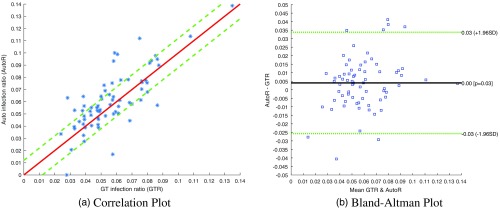
(a) Correlation between automated (y-axis) and manually computed (x-axis) infection ratios for the 70 images of human-NIAID dataset. The straight red line indicates perfect matches. We obtain an average absolute error of 1.18%. (b) The Bland–Altman plot shows the mean signed difference between the automatically computed infection ratio and the manual infection ratio. We report an average error of 0.4% with RPC=0.03 [reproducibility coefficient (1.96×SD)].

Figure 9 shows a comparison of the actual infection ratio with the automatically computed infection ratio based on the SVM classifier output, which we averaged over 10 folds. Figure 9(a) presents the correlation between automated and manually computed infection ratios for the 70 images of human-NIAID dataset. We obtain an average absolute error of 1.18%. Figure 9(b) shows the Bland–Altman plot with a mean signed difference between the automatically computed infection ratio and the manual infection ratio of 0.4%, with RPC=0.03 [reproducibility coefficient (1.96×SD)].

## 6 System Evaluation and Comparison to Commercial Flow Cytometry

**Fig. 10:**
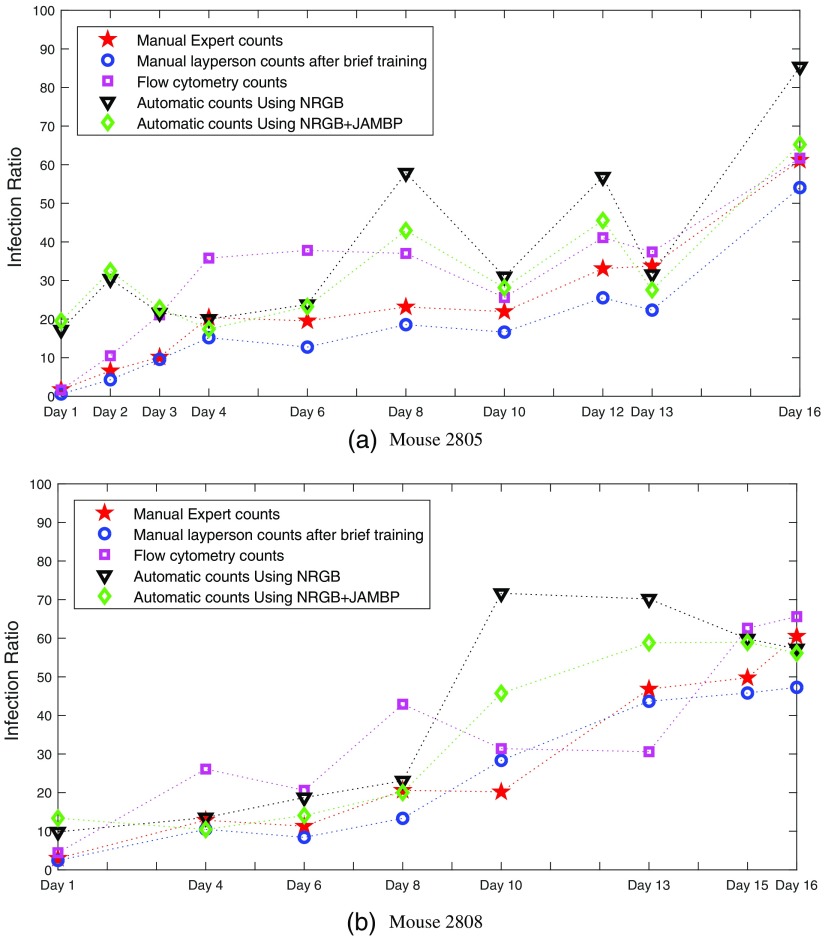
A comparison of automatic counts, using NRGB and JAMBP features, with manual expert counts (red), layperson counts (blue), and flow cytometry counts (pink) for (a) mouse 2805 and (b) mouse 2808.

To evaluate our system systematically, we monitored the malaria infections of two mice identified as 2805 and 2808, during a course of several days. We compared the counts of human experts with the automatic counts provided by our system. In addition, we compared our counts with automatic counts produced by flow cytometry and with the counts of a layperson, who received a brief introduction into the art of cell counting by expert slide readers. Figures 10(a) and 10(b) show these comparisons for mouse 2805 and mouse 2808, respectively. Mouse 2805 has been monitored for 10 days and mouse 2808 for 8 days. In terms of manual counts, Figs. 10(a) and 10(b) show that the layperson’s counts are very close to the expert counts, suggesting that a layperson, after a brief training, can produce about the same quality counts as an expert slide reader. Another observation for Fig. 10 is that there is a noticeable difference between the expert counts and the counts produced by flow cytometry. With a few exceptions, the flow cytometry counts are usually higher than the expert counts. They can be more than twice as high for some days, suggesting that flow cytometry counts and human counts are not interchangeable. However, we observe a strong correlation between manual counts and flow cytometry counts, in particular, for mouse 2805 in Fig. 10. In terms of automatic counts, the NRGB feature performs almost identical to the combination of NRGB and JAMBP for mouse 2805 (black and green curves). However, for mouse 2808, the combination of NRGB with JAMBP outperforms NRGB, as the counts are closer to the expert and flow cytometry counts. We attribute this to the poorer slide quality for mouse 2808, where staining artifacts can more easily lead to FPs when using NRGB alone. The inclusion of texture features, such as JAMBP, helps to discriminate between actual parasites and stain noise. The latter can lead to FPs when using only color features, such as NRGB. Comparing automatic counts with expert and flow cytometry counts, Figs. 10(a) and 10(b) show that our system is over-counting on days 1 and 2 for mouse 2805, and on days 1, 10, and 13 for mouse 2808. However, on the other days, our system is at least as close to flow cytometry as the expert counts, if not closer. Except for the days we are over-counting, the automatic counts are reasonably well correlated with the expert and flow cytometry counts. We again attribute the over-counting to the poor slide quality and staining artifacts, which result in FPs.

## 7 Conclusion

We have developed an image analysis system that can automatically quantify a malaria infection in digitized images of thin blood slides. The system’s image processing pipeline consists of three major steps: cell segmentation, feature computation, and classification into infected and uninfected cells. The most challenging task of the pipeline is the segmentation phase, which needs to be fast and accurate in splitting any clumped cells to avoid miscounting and misclassification in the last stage of the pipeline. We use a combination of multiscale LoG filter and C-EPAC level-set scheme to detect and segment cells, which is capable of identifying individual cells in a clumped cell cluster of touching cells and outperforms other methods. For feature computation, we use a combination of NRGB and JAMBP texture features. The color feature picks up the typical color of stained parasites and the texture feature detects cell texture information including the cytoplasm of parasites. This feature combination works well in our experiments and helps to avoid FPs due to staining artifacts. In the classification step, we evaluate the linear SVM and ANN classifiers performance on human and mouse slides. The ANN classifier achieves F1 score of 90% in correctly identifying infected cells on human-NIAID dataset. We measure an average absolute error of 1.18% between the true and the automatic parasite counts for human. For mouse cells, our automatic counts correlate well with expert and flow cytometry counts, making this the first system that works well for both human and mouse. Compared to human counting, our system is much faster and can process 100  cells/s on low-power computing platforms. The system provides a reliable and standardized interpretation of blood films and lowers diagnostic costs by reducing the workload through automation. Furthermore, the implementation of the system as a standalone smartphone app is well-suited for resource-poor malaria-prone regions. Future image analysis on blood smears could also help in discriminating parasite species and identifying parasite life stages.
